# A Synchronous undifferentiated nasopharyngeal carcinoma and infiltrating ductal carcinoma of the breast successfully treated with induction chemotherapy followed by local control of both tumours: a case report

**DOI:** 10.1186/1472-6815-11-6

**Published:** 2011-06-09

**Authors:** Mohamed Mesmoudi, Tarik Mahfoud, Nabil Ismaili, Khadija Rami, Meryem Kamouni, Laila Jroundi, Hassan Errihani

**Affiliations:** 1 Department of Medical Oncology, National Institute of Oncology, Rabat, Morocco; 2 Department of Radiation Therapy, National Institute of Oncology, Rabat, Morocco; 3 Department of Histopathology, National Institute of Oncology, Rabat, Morocco; 4 Department of Radiology, National Institute of Oncology, Rabat, Morocco

## Abstract

**Background:**

Multiple primary cancers have a low incidence particularly when cancers are synchronous. Few cases of synchronous head and neck cancer and breast carcinoma are reported in the literature.

**Case presentation:**

We report here an exceptional case of a 47 years old Moroccan woman presenting two synchronous cancers, the first in the nasopharynx and the second in the breast. The patient was treated successfully with a combined strategy associating chemotherapy, radiation therapy, and surgery. She remains disease free after 27 months of follow up.

**Conclusions:**

Treatment strategy in the case of multiple primary cancers remains controversial because of the variety of presentations; initial aggressive treatment reports good results.

## Background

The incidence of multiple primary cancers (MPC) is estimated between 0,73% and 11,7% [1]. The association of different cancers is classified in two categories depending on the timing of their discovery; synchronous in which the cancers occur at the same time or within two months, as the case that we report, or metachronous in which the cancers follow in sequence of more than two months apart [2].

The undifferentiated carcinoma of the nasopharynx is a common cancer in North Africa and in the Mediterranean basin, but the incidence of a double malignancy including a nasopharyngeal carcinoma is very uncommon. We report here a case of an undifferentiated nasopharyngeal carcinoma with a synchronous breast cancer treated successfully with induction chemotherapy followed by local control of both tumours.

### Case presentation

A 47 years old Moroccan woman with a familial history of a father dead from a colorectal cancer, and a maternal aunt dead from a breast cancer, presented to our institute with complaints of nasal obstruction, headache, and ear fullness for three months, and skin changes on the right breast for two months. She did not breastfeed her four children, had a regular menstrual cycle, and took oral contraception.

**Figure 1:**
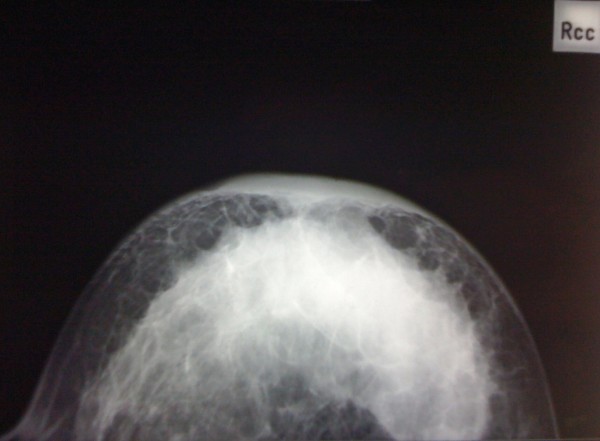
**Breast mammogram**. Right breast mammogram showing an increase in density on the right breast with abnormal skin thickening in the periareolar area and associated microcalcifications.

**Figure 2:**
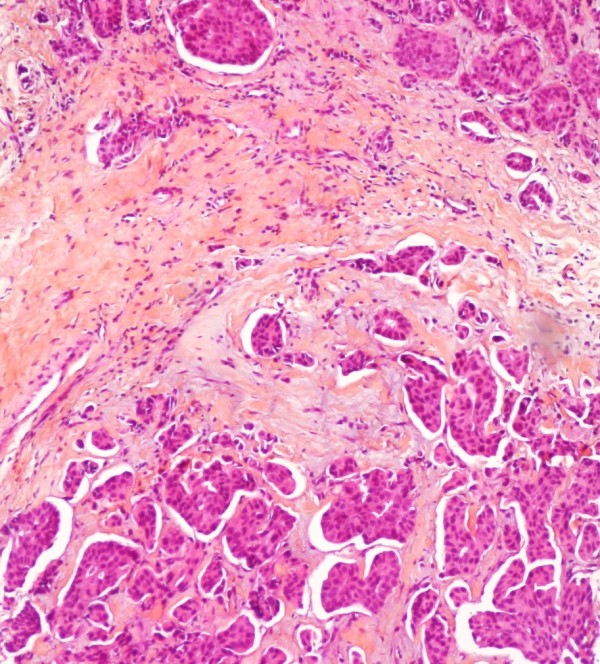
**Histopathology of the breast lesion**. Photomicrograph of the histopathological analysis of the breast biopsy showing an infiltrating ductal carcinoma grade II.

On physical examination, the right breast had a 6 × 5 cm lump on retroareolar area fixed to the skin with thickening of the whole breast and nipple retraction. The axillae and the cervical regions were free of any adenopathy. Mammogram showed an increase in density on the right breast with abnormal skin thickening in the periareolar area and associated microcalcifications (Figure 1), the complement of ultrasound revealed a nodular lesion measuring 4 × 4 cm. On the fine needle aspiration cytology there were signs of malignancy confirmed with core biopsy of the lump suggesting a ductal infiltrating carcinoma with micropapillary component (Figure 2).

**Figure 3:**
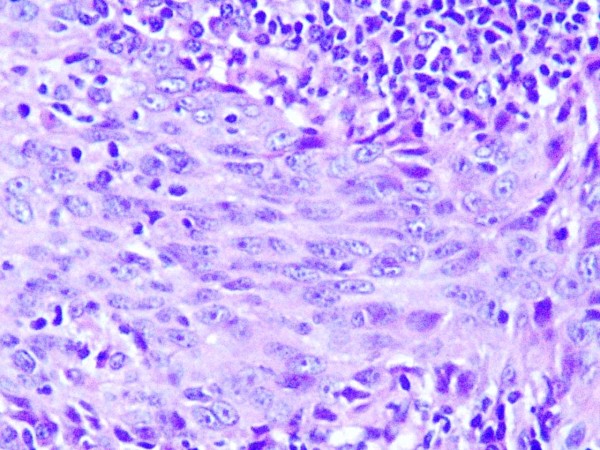
**Histopathology of the nasopharyngeal lesion**. Photomicrograph of the histopathological analysis of the nasopharynx biopsy showing the aspect of an undifferentiated carcinoma.

At presentation our patient had developed a unilateral conductive hearing loss associated with recurrent otitis media and numbness of the face. A computed tomography (CT) of the face and the skull showed an asymmetric mass in the posterior nasopharynx extended into the adjacent parapharyngeal spaces and infiltrating the sphenoid bone, no regional adenopathies were revealed, and the nasopharyngeal biopsy confirmed the diagnosis of an undifferentiated nasopharyngeal carcinoma (Figure 3). There were no distant metastases and we conclude the diagnosis of a locally advanced breast cancer with a synchronous stage III undifferentiated nasopharyngeal carcinoma.

The patient received induction chemotherapy combination of drugs sharing an activity on breast and nasopharyngeal carcinomas, association of taxanes and anthracyclines drugs was used (weekly Paclitaxel 80 mg/m^2 ^for 12 weeks associated to Doxorubicine 50 mg/m^2 ^every 3 weeks) for a total of 4 cycles. The treatment was generally well tolerated; the patient had 3 episodes of grade III-IV non febrile neutropenia, grade II mucositis, total alopecia, and grade II neuropathy reversible after several months thereafter. After the four cycles of chemotherapy there were a remarkable improvement on the breast and a relief in the symptoms of the nasopharyngeal tumour.

The induction therapy was followed by external beam radiation for the nasopharynx (70Gy in 35 fractions) with concurrent chemotherapy (weekly Cisplatine 40 mg/m2 for four weeks) and a prophylactic radiation to cervical lymph nodes. Evolution was marked by the improvement of the symptoms and a radiological reduction volume of the nasopharyngeal mucosa thickening which biopsy confirmed the absence of malignancy. This sequelar lesion has persisted until the last control without trend to progression.

Breast intervention was delayed until after the local control of the nasopharyngeal tumour. A right-sided modified radical mastectomy with axillary nodes dissection was carried out. Histological analysis of the mastectomy specimen revealed a 2 mm residual infiltrating ductal carcinoma grade II, neither *insitu *component nor lymphovascular emboli were found, hormonal receptors were positives (90% on estrogens receptor, and 30% on progesterone receptor), Her2 status was negative, and from the 11 lymph nodes dissected 4 were involved. Surgery was followed by chest wall irradiation, and the patient undergoes now an antihormonal therapy; she has accomplished 12 months of Tamoxifene. The latest medical controls show no signs of locoregional relapse for both tumours and our patient remains disease-free 27 months after beginning therapy.

## Conclusions

The presence of multiple synchronous tumours in the head and neck area and the upper aerodgestive tract is well established [3,4], this association has been explained by the concept of "field cancerization"[5]. In a Chinese analysis 3% of patient with nasopharyngeal carcinoma develop a second cancer, most common site are the aerodigestive tract, lymphoproliferative malignancies, skin, and connective tissue. The risk of developing a second breast cancer was estimated to 1,34 [6].

Sel'chuk *et al *in 100 patients with head and neck cancers found a rate of 19% of synchronous tumours detected on the breast [7]. Strobel *et al *in a systematic detection of synchronous primaries to squamous head and neck carcinomas on 589 patients found one case of breast second cancer [8]. Driss *et al *have reported a case of breast metastases from an undifferentiated nasopharyngeal carcinoma, in the English literature there is only three well documented cases of this setting [9]. The association that we report here includes two primary sites from different histological types.

A retrospective study by Di Martino *et al *concerning the survival in second primary malignancies of patients with head and neck cancers conclude that the prognosis of synchronous tumours is significantly lower when compared to malignancies of a metachronous nature, results report 26% of 5 year overall survival for metachronous setting after the occurrence of the second cancer, and 11,9% for the synchronous setting (p < 0,0001), a second interesting conclusion from this study is that only the early implementation of aggressive treatment methods for second primaries is successful in terms of survival [10]. In our case we have opted for this aggressive strategy and treated our patient with combined modality of chemotherapy drugs which have an activity on the breast and nasopharyngeal cancers followed by local control therapy of both tumours. Results were encouraging with a survival exceeding two years and without local or distant relapse.

The distinctive racial/ethnic and geographic distribution of NPC worldwide suggests that both environmental factors and genetic traits contribute to its development, well-established risk factors for NPC include elevated antibody titers against the Epstein-Barr virus (EBV), consumption of salt-preserved fish, a family history of NPC, and certain human leukocyte antigen class I genotypes [11]. In another hand, an association between two primaries suggests common ethological risk factors.

Positive association between alcohol intake and carcinoma of the breast has been consistently demonstrated [12]. In the case of the nasopharyngeal carcinoma a systematic review by Chen *et al *suggests that heavy alcohol consumption is associated with an increased risk of NPC [13]. Our patient had no history of alcohol intake.

Besides of the exogenous risk factors, Bongers *et al *suggest that an intrinsic susceptibility may influence the risk for the development of second primary tumours in patients with head and neck carcinoma [14], the familial history of cancer in the case that we report might invoke the genetic susceptibility in the pathogenesis of this association of primaries and the hereditary predisposition to multiple cancers.

Other explanation could be the role of EBV which implication is well established like etiological factor of NPC, furthermore, a recent study by Mazouni *et al *provides evidence for EBV-associated breast cancer undergoing distinct carcinogenic processes with more aggressive features [15]. At last, this unusual association of breast cancer and NPC would be due to a chance phenomenon.

Synchronous double malignancy of head and neck and breast is very uncommon, etiology remains obscure, there is no standard approach for the treatment strategy, and in our case treating both malignancies with the same induction chemotherapy combination followed by local therapy was successful.

### Consent

Written informed consent was obtained from the patient for publication of this case report and accompanying images. A copy of the written consent is available for review by the Editor-in-Chief of this journal.

## List of abbreviations

**CT**: Computed tomography; **EBV**: Epstein Barr virus; **MPC**: Multiple primary cancer; **NPC**: Nasopharyngeal carcinoma.

## Competing interests

The authors declare that they have no competing interests.

## Authors' contributions

MM was involved in the analysis of the data, the literature research and wrote the manuscript, TT helped write the manuscript and the literature research, NI helped with modifications and revision of the manuscript, KR approved the radiotherapy part of our patient, MK approved the histopathological part of the case, LJ approved the radiological part of the case, HE managed the patient, approved the treatment and analyzed the literature data. All authors read and approved the final manuscript.

## Pre-publication history

The pre-publication history for this paper can be accessed here:

http://www.biomedcentral.com/1472-6815/11/6/prepub
